# Leading Predictors of COVID-19-Related Poor Mental Health in Adult Asian Indians: An Application of Extreme Gradient Boosting and Shapley Additive Explanations

**DOI:** 10.3390/ijerph20010775

**Published:** 2022-12-31

**Authors:** Mohammad Ikram, Nazneen Fatima Shaikh, Jamboor K. Vishwanatha, Usha Sambamoorthi

**Affiliations:** 1 Department of Pharmaceutical Systems and Policy, School of Pharmacy, West Virginia University, Robert C. Byrd Health Sciences Center [North], P.O. Box 9510, Morgantown, WV 26506-9510, USA; 2 Department of Microbiology, Immunology and Genetics, University of North Texas Health Science Center, Fort Worth, TX 76107, USA; 3 Department of Pharmacotherapy, University of North Texas Health Science Center, Fort Worth, TX 76107, USA

**Keywords:** adult Asian Indians, mental health, COVID-19, machine learning, XGBoost, predictors, sleep disturbance, discrimination, economic crisis, interpersonal trust

## Abstract

During the COVID-19 pandemic, an increase in poor mental health among Asian Indians was observed in the United States. However, the leading predictors of poor mental health during the COVID-19 pandemic in Asian Indians remained unknown. A cross-sectional online survey was administered to self-identified Asian Indians aged 18 and older (N = 289). Survey collected information on demographic and socio-economic characteristics and the COVID-19 burden. Two novel machine learning techniques-eXtreme Gradient Boosting and Shapley Additive exPlanations (SHAP) were used to identify the leading predictors and explain their associations with poor mental health. A majority of the study participants were female (65.1%), below 50 years of age (73.3%), and had income ≥ $75,000 (81.0%). The six leading predictors of poor mental health among Asian Indians were sleep disturbance, age, general health, income, wearing a mask, and self-reported discrimination. SHAP plots indicated that higher age, wearing a mask, and maintaining social distancing all the time were negatively associated with poor mental health while having sleep disturbance and imputed income levels were positively associated with poor mental health. The model performance metrics indicated high accuracy (0.77), precision (0.78), F1 score (0.77), recall (0.77), and AUROC (0.87). Nearly one in two adults reported poor mental health, and one in five reported sleep disturbance. Findings from our study suggest a paradoxical relationship between income and poor mental health; further studies are needed to confirm our study findings. Sleep disturbance and perceived discrimination can be targeted through tailored intervention to reduce the risk of poor mental health in Asian Indians.

## 1. Introduction

The effect of COVID-19 on mental health concerns has been widely researched [1]. In the United States (US), the percentage of adults with depression/anxiety increased from 10.7% in 2019 to 39.2% between April 2020 and April 2021 [2]. During the COVID-19 pandemic, stressors such as social isolation, quarantine, economic concerns, the public health implications of COVID-19, and workplace safety concerns exacerbated poor mental health in the US [3,4,5,6]. Even with COVID-19 vaccine availability, a study using the US Census Household Pulse survey observed that nearly 1 in 4 adults and 1 in 3 adults reported depression and anxiety, respectively [7], and vaccine availability did not change the prevalence of poor mental health [7].

However, it is not known how COVID-19 has affected the mental health of racial and ethnic minorities. Research prior to the COVID-19 pandemic suggests that racial and ethnic minorities such as African Americans, Hispanic/Latinos, and Asians were less likely to have poor mental health compared to Non-Hispanic Whites (NHWs) [8,9,10]. Studies conducted during the COVID-19 pandemic report that NHWs may have poor mental health compared to racial minorities [11]. In another study using data from the nationwide Health Information National Trends Survey (HINTS), it was reported that among those with chronic conditions, COVID-19 was associated with poor mental health among NHWs, and no such association was found among people of color [12].

When examined by racial and ethnic subgroups, Asian Americans experienced the largest increase (560%) in depression/anxiety during COVID-19 compared to pre-COVID-19 [2]. Such an increase in depression/anxiety may be attributed to increased discrimination against Asian Americans during the COVID-19 pandemic [13,14]. It is well-established that discrimination negatively affects the mental health of all ethnic minorities [15,16]. In addition, the high prevalence of poor mental health could also be due to high rates of COVID-19-related morbidity and mortality of family members and relatives in home countries and the inability to support the families during the time of high need because of travel restrictions [17,18].

Asian Americans are not a monolithic group and represent a diverse population from multiple countries, languages, and ethnicity [19]. One of the largest groups among Asian Americans is Asian Indians, who represent a large subset of new migrants in the US [20]. However, little is known about poor mental health and its predictors among Asian Indians in the US during the COVID-19 pandemic. Although research on COVID-19- related mental health among Asian Indians is sparse, conceptual frameworks and published studies of mental health during COVID-19 can guide the selection of potential predictor variables. For example, a narrative review on the epidemiology of COVID-19-related mental health suggested several factors such as age, sex, education, income, stigma, and personal protective measures [21]. Similarly, the determinants of health framework proposed by K. Park include biological, behavioral, socioeconomic, health system, and sociocultural factors to achieve optimal physical and emotional health [22]. It is also suggested that healthy lifestyle behaviors such as enough sleep, adequate nutrition, and other healthy behaviors promote physical and mental health. Furthermore, South Asians have endured discrimination in various forms, exacerbated during the COVID-19 pandemic.

The Asian Indians living in the US have faced long-standing discrimination. External events such as COVID-19 tend to exacerbate discrimination against Asian Indians [23,24,25]. However, Asian Indians have strong cultural identities and often have substantial psychosocial and economic resources that can buffer the effects of stressors [26] and blunt the adverse effects of discrimination on mental health. Many Asian Indians may have become vulnerable to the negative effects of discrimination on mental health because they could not utilize their support systems and economic and psychosocial resources due to COVID-19 restrictions. Thus, our selection of predictors was based on conceptual frameworks, published studies, the unique position of Asian Indians in the US, and the availability of relevant factors in data.

In this context, machine learning (ML) methods are emerging as a helpful tool in epidemiology and health outcomes research [27]. Many ML studies have been conducted to detect and diagnose mental health conditions or symptoms [28,29,30]. Although many ML algorithms can be used for prediction, eXtreme Gradient Boosting (XGboost) has proven to be a highly effective and widely employed technique for prediction in healthcare, including mental health [31,32,33]. Identifying predictors and determining their direction and importance in Asian Indians’ poor mental health is critical for determining future research focus areas. Findings from predictive and explainable ML modeling can inform policymakers and providers to develop targeted efforts to improve mental health at the population and individual levels.

The present study aims to determine the leading predictors of poor mental health among Asian Indians. We additionally used explainable ML to understand the association of predictors with poor mental health during COVID-19 among Asian Indians.

## 2. Methods

### 2.1. Study Design and Data Source

This study used a cross-sectional study design and convenience sampling methods. Using Qualtrics, an online survey was created. Participants had to be 18 years or older and self-identify as Asian Indians. Participants were categorized as Asian Indians if they selected one of the following options: Asian Indians, born in the Indian subcontinent, have origins in the Indian subcontinent, or their parents are from the Indian subcontinent. Flyers and links to the survey were posted in temples, university bulletin boards, and other public places. Institutional review boards at West Virginia University and the University of North Texas approved the survey. The survey was conducted between May 2021 and July 2021.

**Figure 1:**
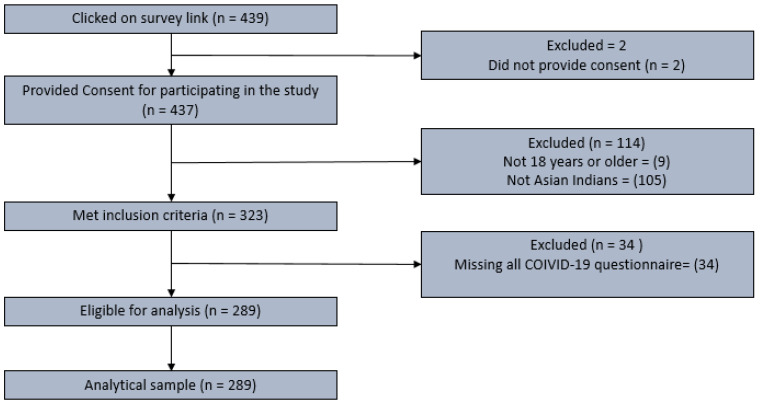
Study sample inclusion flowchart.

Figure 1 provides details of inclusion criteria.

### 2.2. Measures

#### Target Variable: Poor Mental Health (Yes/No)

In the survey, participants were asked about the challenges they faced due to the COVID-19 pandemic. Two of the items in the survey were: (i) feeling down, depressed, or lonely; and (ii) feeling nervous, tense, or worried. We combined the responses of both items to create a binary variable that indicated poor mental health among the participants. If respondents checked one or both of the two items, they were considered to have poor mental health.

Key features: The key features of poor mental health in Asian Indians are presented in Table 1.

### 2.3. Data Preparation and Analysis

The dataset was inspected for missing values, and missing values for age, sex, income levels, and discrimination were imputed. We used the single imputation method and randomly distributed the possible range of the values [39,40]. To prepare the data for analysis using ML methods, we created new binary features from categorical variables using a one-hot encoding process, a common preprocessing technique [41]. The one-hot encoding improves the prediction and classification accuracy of the ML technique. In this study, 70% of the data were used for prediction training, and the other 30% was used as a test dataset. We used the test data to assess model performance.

XGBoost is a widely used ensemble ML technique that combines multiple ML methods for improved predictive accuracy [31]. A decision tree is used as a base learner in XGboost, and new decision trees are added to the ensemble process [42]. XGboost combines many weak learners and subsequently makes a strong learner [31,42]. It is a highly flexible method that allows one to tune many in-built hyperparameters [43,44]. It also uses loss function, regularization, and randomization that increase computational speed and predictive accuracy [43].

Stratified 10-fold cross-validation was used for the training dataset, and hyperparameter tuning was used for performance optimization. In order to correct for any imbalance in the distribution of the targeted variable, we additionally employed scale_pos_weight as one of the hyperparameters. Accuracy (percent of poor mental health predicted correctly), precision (poor mental health predicted correctly divided by poor mental health prediction), recall (poor mental health predicted correctly divided by poor mental health cases), F1 score (an average of precision and recall), and area under the curve (AUROC) were among the performance metrics used to assess the classification of the target variable.

### 2.4. Interpretable XGboost

XGboost, like many other ML methods, provides emphasis on prediction [45]. The complexity of predictive models has led to the development of interpretable ML methods. These models identify the variables influencing a prediction [45] and produce summary statistics, which can be used to interpret the association of features with the target variable [45]. Another important aspect of interpretable machine learning is the visualization tools that provide insight into the direction and importance of predictors in the model [45].

For interpreting the results of the XGboost models, we used a model-agnostic ML technique called Shapley Additive exPlanations (SHAP). SHAP is a popular ML technique for gaining insight into the complex relationship between features and prediction [46]. SHAP calculates the contribution of each feature by computing SHAP values and provides the distribution of the predictions among the features. Therefore, the SHAP models can be used to examine how changes in feature distribution will affect the model’s output.

SHAP results can be explained in interpretable components. The two most commonly used approaches to explain SHAP results are (1) global and (2) local interpretation [47]. A global interpretation summarizes the contribution of each feature to the prediction. In the process, global interpretation can plot features positively and negatively to demonstrate their directionality with the target variable. For global interpretability, feature importance, summary plot, and partial dependence plots are used. In the local interpretation, SHAP values are used to explain the contribution of each feature for a single observation. In local interpretation, each observation is assigned a model score based on the contribution of features to that score. Local interpretability is based on individual SHAP value plots. Global and local SHAP plots were generated using TreeSHAP. In addition to predictions and interpretation of each feature, we also analyzed interaction effects. For SHAP interaction plots, we used the Xgbfir package. The ML methods were performed using Python 3.8.8. For survey data cleaning and initial variable setup, we used SAS 9.4.

## 3. Results

A majority of the study participants were below 50 years of age (73.3%), female (65.1%), had at least a college degree (84.4%), and belonged to an income level of ≥$75,000 (81.0%). Their general health scores ranged from 36 to 100 (mean = 82.7, SD = 14.0), where a lower score indicates poor general health and a higher score indicates better general health. Nearly 66% of Asian Indians reported some form of discrimination. Sleep disturbance was reported by 20% of the participants. Among the 289 participants, 133 (46%) Asian Indians reported poor mental health.

Model Performance: The model performance metrics are presented in Table 2.

Table 2: Model Performance Metrics Using Test data; Poor Mental Health in Asian Indians aged 18 years and older.

**Figure 2:**
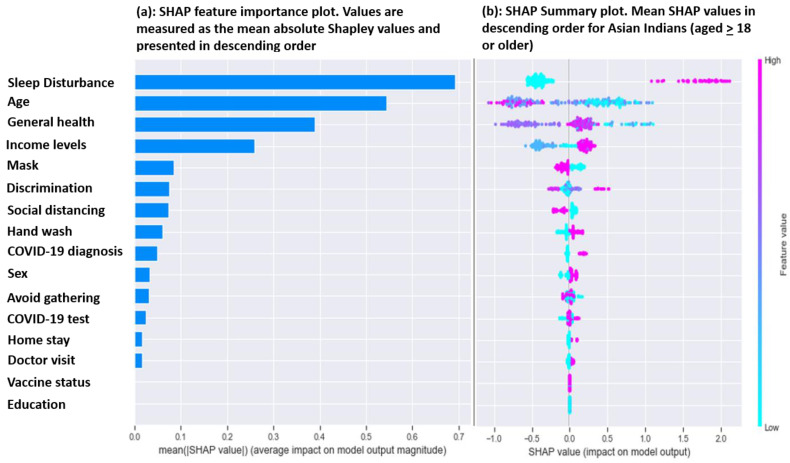
Feature importance and SHAP summary plot for COVID-19-related poor mental health in Asian Indians. The model is based on 289 Asian Indian adults (age ≥ 18). The *X*-axis represents the marginal contribution of a feature to the change in the predicted probability of poor mental health. Feature values represent the actual value of the predictors (e.g., values for age will range from 18 years to 96 years; values closer to 18 years were represented with blue dots, and values closer to 96 were represented with purple).

Feature importance: We present the key predictors of poor mental health among Asian Indians in Figure 2a using a feature importance plot derived from SHAP values. The higher SHAP value in Figure 2a represents a more important contribution to the poor mental health model. In our study, the top five leading predictors were (1) sleep disturbance, (2) age, (3) general health, (4) income levels and (5) mask.

### Feature Association

Global Interpretation: The directionality and importance of predictors in relation to the poor mental health of Asian Indians are presented in Figure 2b. Predictors are ordered from most to least important. In the plot, each dot represents an individual. Each feature represents the distribution of its impact on the poor mental health of Asian Indians. SHAP values of numeric features are represented by pink and blue colors, where pink is attributed to larger values and blue to smaller ones. The number of examples at a given value determines the thickness of the line. A negative SHAP value indicates a lower risk of poor mental health, and a positive value indicates a higher risk of poor mental health. The plot shows that adults with sleep problems and a COVID-19 diagnosis are at a higher risk of having poor mental health. Conversely, wearing a mask and maintaining a 6-feet distance reduced the likelihood of poor mental health.

**Figure 3:**
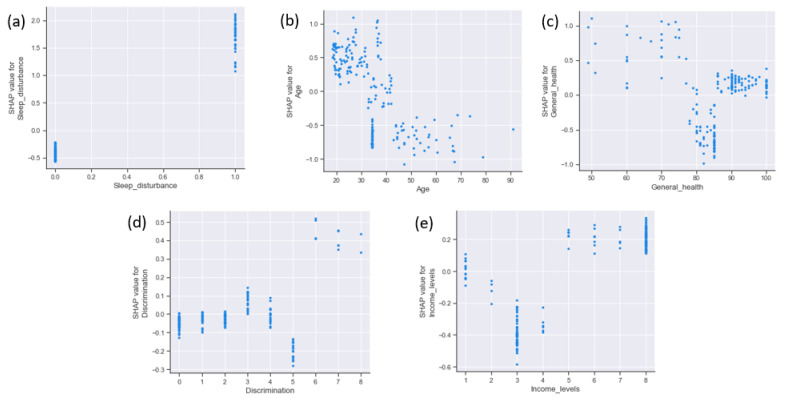
Shapley additive explanation (SHAP) dependence plots of key features in the poor mental health model among Asian Indians aged 18 years and older. Note: Each point on the plot corresponds to a prediction made for an Asian Indian. The SHAP dependence plot shows the main effects of features on poor mental health by (**a**) sleep disturbance, (**b**) age, (**c**) general health (**d**) discrimination and (**e**) income levels.

In our analysis, higher values of age were associated with lower SHAP values and lower values of age were associated with higher SHAP values. Having a sleep disturbance was associated with higher SHAP values. In both cases, higher values indicate a positive association with poor mental health, and lower values indicate a negative association with poor mental health. The directionality of the summary plot was mixed for some features. Higher values for general health were distributed from negative to positive SHAP values. The directionality of discrimination to mental health was mixed. Higher discrimination values were distributed on both the positive and negative sides of the SHAP values. Therefore, to gain a clear picture of directionality, we created a SHAP dependency plot (Figure 3). From 0 to 4, the SHAP values for discrimination were mostly neutral. For values 6 and above, SHAP values went up sharply. For the feature age, SHAP values were higher at lower ages (20 to 30 years old). For age values of 45 and over, SHAP values were mostly negative. The SHAP dependency plot revealed that sleep disturbance (value = 1) was directly associated with a higher SHAP value and no sleep problem (value = 0) was associated with a negative SHAP value. General health scores, approximately between 60 and 75, indicated higher SHAP values, while values of 80 and over were associated with negative to positive SHAP values.

**Figure 4:**
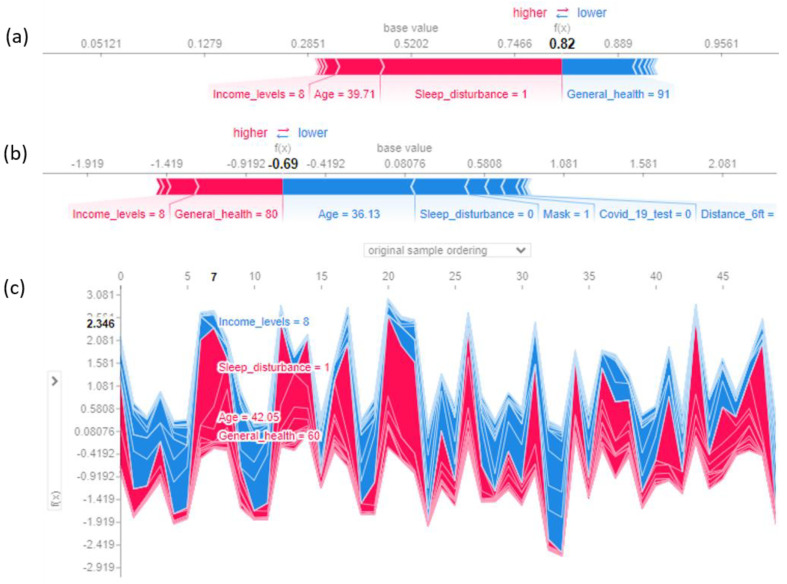
Shapley force plots of individual and collective observations. (**a**,**b**): Features in red influence the model towards poor mental health, and features in blue influence the model away from poor mental health. The size of the features represents their importance (for example, the most important feature in (**a**) was sleep disturbance). Features close to the red and blue color separation marks have a greater impact on the model. (**c**) represents the collective force plot of 50 individuals. In the collective force plot, the *Y*-axis is the *X*-axis of the individual force plot.

Local interpretation: Figure 4 represents the effects of features on the individual and collective observations. Features in red influence the model towards poor mental health, and features in blue influence the model away from poor mental health. The number in bold represents the model score for a particular observation. The size of the features indicates their importance. For example, in Figure 4a, sleep disturbance is the most important feature driving the model to a higher score. Features close to the red and blue color separation marks have a greater impact on the model. Figure 4a shows how red features, such as sleep disturbance and age, influence the model to a higher score. Therefore, interventions aimed at improving sleep disturbance may push the model to a lower score. Figure 4b shows that age (36 years) and no sleep disturbance influence the model toward a negative score, whereas general health (score = 80) pushes toward a higher score. Therefore, for further improvement, intervention may target improving general health. Figure 4c represents the collective effects of all features for the first 50 observations by original sample order. The figure shows that for observation no. 7, age (42 years), sleep disturbance, and poor general health (score = 60) are driving the score towards positive prediction (poor mental health).

Table 3 depicts key features interactions and their effects on poor mental health. Gain, F1 score, and weighted F1 score, along with their position in the model, are provided. General health and sleep disturbance had the highest gain score of 182.9 with a gain rank of 1 in the model.

## 4. Discussion

In this study, we identified the leading predictors of poor mental health in Asian Indians in the US by using ML methods. This study expands the current knowledge of COVID-19-related mental health among Asian Indians and contributes uniquely by identifying key predictors. In this study, the leading predictors of poor mental health were: sleep disturbance, age, general health, income levels, wearing a mask all the time, discrimination, and practicing social distancing all the time. In our study, nearly half of the Asian Indians (46.2%) reported poor mental health. This percentage is slightly higher than the percentage found by Lozano et al. (2022) who reported that the prevalence of depression in South Asians in Chicago was 38% [48].

In our study, 20% of Asian Indians reported sleep disturbance. This finding is consistent with the findings of the Mediators of Atherosclerosis in South Asians Living in America (MASALA) study [49], which found that 24% of South Asians are at high risk for obstructive sleep apnea. It is not surprising that sleep disturbance was the leading predictor of poor mental health. Many studies, not specific to Asian Indians, have confirmed sleep disturbance as one of the common comorbidities of poor mental health, such as anxiety and depression [50,51,52]. However, the relationship between sleep disturbance and poor mental health could be bidirectional [53]. Nonetheless, longitudinal studies have indicated that sleep problems, particularly insomnia, as a risk factor for depression [50,54]. During COVID-19, an increase in sleep disturbances was observed [55,56], which may have contributed to poor mental health. We also cannot rule out the complex relationship between sleep disturbance, acculturation, discrimination, and mental health [15,57,58,59]. The American College of Physicians recommends cognitive behavioral therapy (CBT-I) for all adults with insomnia [60]. However, several factors, such as acculturation, lack of knowledge about treatment options, monetary issues, English proficiency, and reliance on children for transportation by older adults, may limit their ability to seek CBT-I [58,61].

Age was the second leading predictor; the partial dependency plot demonstrates that being younger adults was associated with a positive prediction of poor mental health during the COVID-19 pandemic, which is consistent with the literature [62,63,64]. Studies have cited that greater concern over the future and economic conditions could be the reason for higher levels of depression and anxiety in lower age groups [65,66]. Another reason for higher depression could be the excessive consumption of COVID-19-related news by young adults [67]. Olagoke et al. (2020) reported that increased consumption of COVID-19-related news was associated with depression symptoms [68]. As media consumption decreases with age, older age protects against higher media consumption and increases higher behavioral media avoidance compared to younger age [67].

General health was the third leading predictor of poor mental health. In our study, a lower general health rating was associated with poor mental health. Many studies have reported that subjective health ratings could affect mental well-being [69,70,71,72]. Although a lower subjective health rating is generally associated with poor mental health [69,70,71,72], studies have documented the complex and reciprocal relationship between physical and mental health [73,74,75]. However, causal and mediatory analysis suggest that physical activity and social interactions can improve both mental and physical health [76]. As the COVID-19-related mental health burden is expected to last long after COVID-19 [77,78], health interventions to reduce the risk of poor mental health need to focus on physical activity and social interactions [76,79].

Income levels were the fourth leading predictor of poor mental health. A paradoxical relationship between income and poor mental health was observed. Compared to the lower income levels, higher income levels were associated with poor mental health. This contrasts with some published studies that report an association between low socioeconomic status and poor mental health [80]. However, studies on the relationship between income and poor mental health suggest varying relationships among different racial groups. For example, African American men with high incomes were more likely to report poor mental health [81,82]. We speculate that the composition of social networks among high-income Asian Indians may be different and add to the stress leading to poor mental health. The COVID-19 pandemic led to a major economic crisis in the US, and that could have affected the relationship between mental health and income for an ethnic minority such as Asian Indians. Research has shown that during the economic crisis, interpersonal trust was the only significant protective factor for mental health [83,84]. Asian Indians have a robust culture of strong social relationships that are based on interpersonal trust [85]. Such interpersonal trust becomes even more important in challenging times such as the COVID-19 pandemic. However, the COVID-19 pandemic led to social/interpersonal isolation, reducing family support and social networks, and ultimately affecting mental health globally as well as in specific ethnic groups, including Asian Indians. This may partly explain the paradoxical relationship between income levels and poor mental health among Asian Indians living in the US. We also cannot rule out that the paradoxical relationship may be due to small cell sizes across income and discrimination scores and the use of an ordinal scale to measure income levels. Future studies with robust study designs are needed to confirm this relationship.

COVID-19-related preventative behaviors such as wearing a mask (rank 5), and practicing social distancing (rank 7) were two important predictors of poor mental health. Our finding suggested that wearing a mask and practicing social distancing were protective against poor mental health, consistent with previous studies [86,87]. However, some studies also indicated that social distancing and staying home may have exacerbated poor mental health [3,88,89]. We are unable to determine the causal impact of preventive behaviors on mental health due to our cross-sectional study design.

Self-reported discrimination was the 6th leading predictor of poor mental health in Asian Indians. In our analysis, the dependency plot of discrimination and poor mental health showed that higher discrimination levels (6–8 points of accumulated discrimination) were positively associated with poor mental health. Even before the COVID-19 pandemic, South Asians were subjected to major and everyday discrimination [90]. Such discrimination may cause Asian Indians to experience elevated levels of anxiety and guilt/shame [59], resulting in poor self-reported health [90]. During the COVID-19 pandemic, higher rates of discrimination against Asian Americans, including Asian Indians, have been reported [91] and it is well-established that discrimination negatively affects mental health [15,16].

The current study did not collect data on family structure, psychosocial resources, or religious and spiritual beliefs. We note that a cultural lens needs to be applied in examining and understanding mental health among Asian Indians. We speculate that culture may either provide positive coping and become a source of strength or the acculturation challenges add to the stress and negatively affect mental health. Furthermore, in Indian culture, negative emotions are not freely expressed because they are at times associated with karma and past sins. As Indian culture is based on collectivism rather than individualism, mental health may also be associated with physical symptoms [92]. Therefore, there is a need for future studies to incorporate variables that provide a cultural perspective to explain and reduce poor mental health among Asian Indians. As we anticipate COVID-19-related mental health problems to persist for a long time, considerations for policy and practice must recognize the cultural influences on mental health and provide integrated and holistic approaches to reduce the burden of mental health in specific ethnic groups [93].

This study has several limitations. We used convenience sampling, and a comparison with the 2018 National Health Interview (not presented here) suggested that our study sample is more likely to be female and younger, which may have affected the rates of poor mental health observed in our study. We could not reliably assess the association of vaccination status with poor mental health because 90% of the respondents had received at least one dose of the COVID-19 vaccine at the time of the survey. In addition, the study population is Asian Indian, hence, the findings from this study may not be generalizable to the general population. This study also has several strengths. This is the first study that has identified the leading predictors of poor mental health related to COVID-19 in Asian Indians. Several of these predictors of poor mental health are modifiable factors that can assist in developing targeted interventions to combat increasing mental health concerns. We used novel interpretable ML methods to identify the leading predictors and their associations with poor mental health.

## 5. Conclusions

In this study, nearly one in two Asian Indians reported poor mental health. Sleep disturbance, discrimination, income level, and COVID-19-related preventative behaviors were some of the modifiable and leading predictors of poor mental health in Asian Indians. These findings point to the importance of improving sleep and providing resources for social support among Asian Indians in order to reduce the risk of poor mental health. Similarly, ongoing activities that incorporate diversity and campaigns to reduce stereotypes and prejudice at local, regional, and national levels may be needed to reduce the risk of poor mental health due to discrimination.

## Figures and Tables

**Table 1:** Description of Features.

Feature Name	Feature Levels	
	Coded as 0 (Count)	Coded as 1 (Count)
Sex		
	Male = 101	Female = 188
Education		
	College = 244	LT College = 45
Mask		
	Not all the time = 109	All the time = 180
Hand wash		
	Not all the time = 138	All the time = 151
Social distancing		
	Not all the time = 199	All the time = 90
Avoid gathering		
	Not all the time = 125	All the time = 164
Stayed home		
	Not all the time = 254	All the time = 35
Doctor visit		
	Within 12 months = 193	Other = 96
COVID-19 test		
	No = 112	Yes = 177
COVID-19 diagnosis		
	No = 256	Yes = 33
Sleep disturbance		
	No = 231	Yes = 58
Vaccine status		
	No = 29	Yes = 260
Age		
	Range 18–96	Continuous
Discrimination *		
	Range 0–8	
General health		
	Range 36–100	Continuous
Income levels *		
	Range 1–8	

Note: Based on 289 Asian Indian adults aged 18 or older. *Discrimination was created using three questions that inquired about whether participants were “treated with less respect”, “threatened or harassed”, and “made feel like an outsider”. Each question has a range between 0 and 4, where a higher number represents greater discrimination. Therefore, the possible range for discrimination is 0–12. In our sample, the highest value was 8. * Income levels represent annual household income. The lowest level 1 represented income less than $15,000, and level 8 represented income $100,000 and above. General health represents a health rating scale ranging between 0 and 100. We have imputed data for missing values of age, sex, discrimination, and income levels. Social distancing was defined as maintaining a 6-ft distance. The preventative behaviors questions were adopted using COVID-19 everyday prevention actions from the Centers for Disease Control and Prevention [34]. The discrimination questions were adopted from two different sources [35,36,37,38].

**Table 2:** XGboost Model Performance.

Metrics	Metrics Scores (Weighted Average)
Accuracy	0.77
Precision	0.78
Recall	0.77
F1 Score	0.77
AUROC	0.87

Note: Based on 289 samples. Test data consisted of a 30% random sample of the original data.

**Table 3:** Key Feature Interactions in the Poor Mental Health Model among Asian Indians Aged 18 Years and Older.

Feature Interactions	Gain	F1 Score	Weighted F1 Score	Gain Rank	F1 Score Rank	Weighted F1 Score Rank
General health and sleep disturbance	182.9	20	10.0	1	3	2
Age and general health	177.7	25	7.3	2	1	4
Age and age	171.7	25	8.6	3	2	3
Age and Income levels	166.2	14	6.4	4	5	5
Age and sleep disturbance	157.4	5	4.0	5	7	6
general health and general health	97.9	17	10.2	6	4	1
Age and mask	76.0	10	3.0	7	6	9
Income levels and sleep disturbance	50.5	4	3.2	8	11	8
Age and hand wash	44.8	4	0.9	9	12	19
Age and avoid gathering	31.3	3	0.6	10	16	28

Note: Based on 289 Asian Indians.

## Data Availability

The data associated with the study are not publicly available but are available from the corresponding author upon reasonable request.
